# Resident primary care practitioners’ awareness and handling of zoonotic diseases: an explorative online survey in the Hameln-Pyrmont district, Lower Saxony, Germany

**DOI:** 10.1186/s12875-025-02918-7

**Published:** 2025-08-08

**Authors:** Michael W. Scheider, Lothar Kreienbrock, Thomas von Lengerke

**Affiliations:** 1 Department of Medical Psychology, Center of Public Health and Healthcare, Hannover Medical School, Carl-Neuberg-Str. 1, Hannover, 30625 Germany; 2 Institute for Biometry, Epidemiology and Information Processing, University of Veterinary Medicine Hannover, Hannover, Germany

**Keywords:** Zoonoses, Primary care practitioners, Outpatient healthcare, (Diagnostic) uncertainty, (Diagnostic) confidence, One health

## Abstract

**Background:**

Primary care practitioners often are the first medical professionals to see patients with zoonoses. So far, studies on awareness and management of zoonoses in primary care have focused on specific zoonoses, most prominently Lyme disease. Additionally, (diagnostic) uncertainty vs. confidence of this group of practitioners regarding zoonoses has rarely been examined. Finally, little is known about zoonoses in primary care in Germany. This study aims to describe German resident primary care practitioners’ awareness and handling of zoonoses, and their confidence regarding diagnostics, therapy, and transmission.

**Methods:**

A cross-sectional online survey of primary care practitioners in the Hameln-Pyrmont district, Germany, was conducted between November 6, 2022-January 5, 2023 via regional physician associations. Items on practitioners’ awareness and handling of zoonoses in practice were designed to fit the study’s aims, and explicitly excluded coronavirus disease 2019 (COVID-19). Data were analyzed descriptively and by Wilcoxon signed-rank tests and t-tests for paired samples.

**Results:**

*N* = 43 of the 88 practitioners in the district took the survey (response rate: 48.9%). Zoonoses were seen as more relevant than for the time period before COVID-19 (*p* < 0.001). Among the up to three zoonoses respondents could mention to occur in practice, borreliosis (21 of 98 mentions overall), salmonellosis (10) and toxoplasmosis (7) were named most often. Practitioners’ confidence ratings on diagnostics, therapy, and transmission of zoonoses were higher for self-reported zoonoses than for zoonoses in general (with few exceptions, differences were statistically significant: *p* ≤ 0.030). Confidence was higher for transmission than for diagnostics and therapy for self-mentioned zoonoses and zoonoses in general (*p* ≤ 0.012). Ratings for diagnostics and therapy did not show statistical significance. Almost two thirds of the respondents (64.7%) did not know the term “One Health”.

**Conclusions:**

Borreliosis appears to be the zoonosis with the highest level of attention in primary care. Results on confidence regarding diagnostics and therapy indicate capacities in terms of relatively high confidence regarding salient zoonoses, and room for improvement signified by the lower confidence regarding diagnostics and therapy than transmission. Awareness of uncertainties regarding zoonoses in primary care may trigger continuing medical education, cooperation between medical doctors and veterinarians, and One Health implementation.

**Supplementary Information:**

The online version contains supplementary material available at 10.1186/s12875-025-02918-7.

## Background

Zoonoses are infectious diseases caused by pathogens that are naturally transmissible between vertebrate animals and humans [1]. They represent an increasingly important and widespread threat to global health [2]. Primary care practitioners often are the first medical professionals to see patients with (symptoms of) a zoonotic disease. For instance, the prevalence of Lyme disease, which is caused by borrelia bacteria, after tick bites in Germany has been estimated to be 20% [3]. Since the first worldwide severe acute respiratory syndrome coronavirus (SARS-CoV-1) outbreak, studies on the diagnosis and management of zoonoses in primary care have primarily focused on Lyme disease [4–24]; one study each examined Campylobacter and Salmonella [25] and toxoplasmosis [26]. In Germany, over and above one case report on Lyme arthritis [27] and a pre-SARS-CoV-1 report on salmonella control from the viewpoint of a regional public health office [28], little is known about the practices of resident primary care practitioners regarding patients with (symptoms of) zoonotic diseases. At the same time, (diagnostic) uncertainty vs. confidence, an issue with relevance for clinical decisions [29, 30], has not been examined in the context of zoonoses in primary care. Thus, this study aims to provide insights both into resident primary care practitioners’ awareness of zoonoses and their medical care for zoonotic patients in Germany, and into self-reported confidence of these practitioners regarding diagnostics, therapy, and transmission routes of zoonoses.

## Methods

### Design and setting of the study

A cross-sectional online survey of all 88 resident primary care practitioners in the Hameln-Pyrmont district in the state of Lower Saxony, Germany, was conducted between November 6, 2022, and January 5, 2023, using the online survey software SoSci Survey. The survey link was e-mailed to the practitioners by the regional physician associations via the Medical Association of Lower Saxony. After clicking on the link, information was provided to the practitioners that participation was voluntary and ensured anonymity, that they were taking part in a scientific study, and that they were able to refuse or withdraw their participation at any time. With this procedure, informed consent to participate was obtained from all of the participants in the study in accordance with national regulations. Reminders were sent after two weeks and subsequently by a regional social media group of physicians in the district. The study was approved by the Ethics Committee of Hannover Medical School on October 28, 2020 (vote no. 9419_BO_K_2020).

### Measures

The survey questionnaire used was developed specifically for this study (an English language version is provided in the Supplementary file to this manuscript). Items were specifically designed to fit the aims of the study. The first item assessed whether at least one zoonosis besides coronavirus disease 2019 (COVID-19) was known to the participant; affirming this defined eligibility to continue the survey. Socio-demographic items on sex and age, which followed standards of the German Federal Statistical Office (Destatis) [31], were placed at the end of the survey together with five items on years of professional experience, type of practice (single vs. group/shared), residential area where the practice was located (urban vs. rural), and number of patients per quarter.

Substantive items included an item each for the relevance of zoonoses in one’s practice before and after the start of the pandemic (Likert-item scaled „1 not relevant at all” to 5 “very relevant”). Using an open format, participants were asked to name up to three zoonoses they had already encountered in their practice (again excluding COVID-19). For each of these zoonoses, and for zoonoses in general, participants rated their confidence regarding diagnosis, therapy, and transmission routes on Likert-items scaled „1 very low” to 5 “very high”. Other items assessed whether zoonoses had been an explicit topic in one’s medical curriculum (yes/no); whether any continuing medical education had been taken in the last ten years (yes/no), and if yes, how many courses (1–2, 3–5, or 6 or more); whether they treated zoonoses by themselves mostly (vs. referring them to others) vs. depending on type of pathogen, severity of symptoms, and complexity of therapy vs. never; how many zoonoses they estimated to occur in their practice per year; whether they counseled patients on preventing or treating zoonoses never, sometimes or often; whether they knew where up-to-date information on zoonoses is available, and whether information material is made available to patients in the practice; whether there ever had been any cooperation with a veterinarian in the context of a zoonotic treatment; and whether they knew the term “One Health”. Finally, another set of items referred to pets: respondents were asked whether they questioned patients about pet ownership at all, and if yes, at what instances (at first admission, symptom-driven, and/or at other occasions, e.g. regarding preventive interventions); how many of their patients they estimated to be pet owners; and how many patients they estimated to have regular contact with pets, wild animals, and farm animals.

### Statistical analysis

Data were exploratively analyzed by frequency counts and other descriptive statistics, and regarding differences in confidence ratings by Wilcoxon signed-rank tests and t-tests for paired samples. A power analysis was omitted due to the explorative aims of the study and its full survey approach in regard to all 88 primary care practitioners working in the district at the time of the survey.

## Results

### Sample composition

Table 1 describes the sample of physicians participating in the survey. Of the 88 resident primary care practitioners, 47 accessed the survey site, and *N* = 43 started the survey, which represents a response rate of 48.9%. Three participants indicated to be unaware of any zoonosis besides COVID-19, which by design ended the survey. Of the remaining 40 participants, six discontinued the survey before the socio-demographic items placed at the end of the survey. Thus, the sample description by socio-demographic variables in Table 1 is based on *N* = 34, while the following analyses incorporate the other six participants.

**Table 1:** Sample characteristics

Description	Category	*N*	%
Population of resident primary care practitioners in the district		88	100%
* Accessed survey*	No	41	46.6%
	Yes	47	53.4%
* Started survey (i.e., participated)*	No	45	51.1%
	Yes	43	48.9%^$^
Sample of participating resident primary care practitioners		43	100%
* No zoonosis known, i.e., not eligible for continuation of survey*	No	3	6.9%
* At least one zoonosis known, i.e., eligible for continuation of survey*	Yes	40	93.1%
At least one zoonosis known, i.e., eligible for continuation of survey		40	100%
Discontinued survey anywhere between question on knowledge of any zoonosis and socio-demographics^*^		6	15.0%
Completed survey including socio-demographics^*^		34	85.0%
Completed survey including socio-demographics^*^		34	100%
* Gender*	Men	20	58.8%
	Women	14	41.2%
* Age (in years)*	31–40	1	2.9%
	41–50	11	32.4%
	51–60	17	50.0%
	> 60	5	14.7%
* Professional experience (in years)*	< 11	5	14.7%
	11–20	21	61.8%
	> 20	8	23.5%
* Type of practice*	Single practice	9	26.5%
	Group or shared practice	25	73.5%
* Residential area of practice location*	Rural	15	44.1%
	Urban	19	55.9%
* Number of patients in practice (per annum)*	500–750	1	2.9%
	751–1000	1	2.9%
	1001–1250	2	5.9%
	1251–1500	4	11.8%
	1501–1750	7	20.6%
	> 1750	19	55.9%

^$^Survey response rate

^*^Placed at the end of the survey

There were more men (58.5%) than women among the practitioners, and half of the sample were in the age range of 51 to 60 years (> 60 years: 14.7%). Over 60% had 11 to 20 years of professional experience (> 10 years: 23.5%), and almost three quarters worked in a group or shared practice (73.5%). Finally, a majority of practices were located in urban areas (55.9%), and cared for more than 1.750 patients per quarter (55.9% as well).

### Awareness and handling of zoonotic diseases

As Table 2 shows, retrospectively for the time period preceding the start of the COVID-19 pandemic, 50% of the respondents used the rating “2” on the 5-point Likert-item for the relevance of zoonoses (no one used “5” for “very relevant”), whereas the mode value for the time of the survey, i.e., the final phase of the pandemic, was “5” with 39.5% (means: 2.4 vs. 3.9; medians: 2 vs. 4; *p* < 0.001). Respondents estimated the annual number of zoonotic cases in their practice anywhere between 0 and 50, with the distribution’s mode located at 10, the median at 15, and the mean at 19.5. While 63.2% reported that zoonoses had been an explicit topic in their medical curriculum (34.2% did not know), almost two thirds (65.8%) had taken no continuing medical education on the topic in the last ten years (don’t know: 13.2%). Almost as many (64.7%) did not know the term “One Health”.

**Table 2:** Awareness and handling of zoonotic diseases: descriptive results^*^

Description	*N*	%
Relevance of zoonoses in respondent’s practice		
* before the COVID-19 pandemic*		
Not relevant at all (1)	3	7.9
(2)	19	50.0
(3)	13	34.2
(4)	3	7.9
Very relevant (5)	0	0
* after the COVID-19 pandemic*		
Not relevant at all (1*)*	1	2.6
(2)	3	7.9
(3)	9	23.7
(4)	10	26.3
Very relevant (5)	15	39.5
Zoonoses had been explicit topic in one’s medical curriculum		
I don’t know	13	34.2
No	1	2.9
Yes	24	63.2
Any continuing medical education taken in the last ten years		
I don’t know	5	21.1
No	25	65.8
Yes	8	13.2
* If yes: 1–2*	*3*	*37.5*
* If yes: 3–5*	*3*	*37.5*
* If yes: 6 or more*	*2*	*25.0*
Term “One Health” known		
* No*	22	64.7
* Yes*	12	35.3
Ever counseling on zoonoses		
* for prevention*		
* No, never*	4	11.8
* Sometimes*	26	76.5
* Yes, often*	4	11.8
* for treatment*		
* No, never*	3	8.8
* Sometimes*	28	82.4
* Yes, often*	3	8.8
Zoonoses treated mostly oneself, depends, or never oneself		
* Never oneself*	0	0
* Depends*	26	74.3
* Mostly oneself*	9	25.7
Aware of where up-to-date information of zoonoses can be obtained		
* No*	4	11.8
* Yes*	30	88.2
Up-to-date information on zoonoses made available for patients in practice		
* No*	30	88.2
* Yes*	4	11.8
Ever ask patients about pet ownership (at all?)^**^		
* No*	2	5.7
* Yes*	33	94.3
* If yes: at first admission*	*7*	*21.2*
* If yes: symptom-driven*	*32*	*97.0*
* If yes: at other occasions*	*9*	*27.3*
Ever cooperated with a veterinarian on treatment of a zoonosis		
* No*	28	82.4
* Yes*	6	17.6

^*^Different totals of N’s across variables due to different number of item missings

**For sub-categories of "yes", multiple responses were possible

As Table 2 further shows, around 10% of the respondents never counseled patients on zoonoses (11.8% regarding prevention and 8.8% regarding therapy), and almost three quarters (74.3%) treated zoonoses autonomously (vs. referring them) only depending on pathogen type, symptom severity, and complexity of therapy. Any cooperation with a veterinarian in the context of a zoonotic treatment was reported by 17.6%. Most practitioners (94.3%) reported to ask patients re pet ownership. Not shown, on average they estimated that 32.1% of their patients owned a pet (range: 5–65, with both mode and median at 30), and 43.9% have regular pet contact (range: 5–80, mode: 30, median: 50; the corresponding estimates for wild and farm animals were 7.8%, 1-100, 5, and 2, and 10.9%, 1–70, 10, and 8, respectively). *N* = 30 (88.2%) reported to know where information on zoonoses were available, but only *N* = 4 (11.8%) that such material was made available to their patients in their practice.

**Fig. 1:**
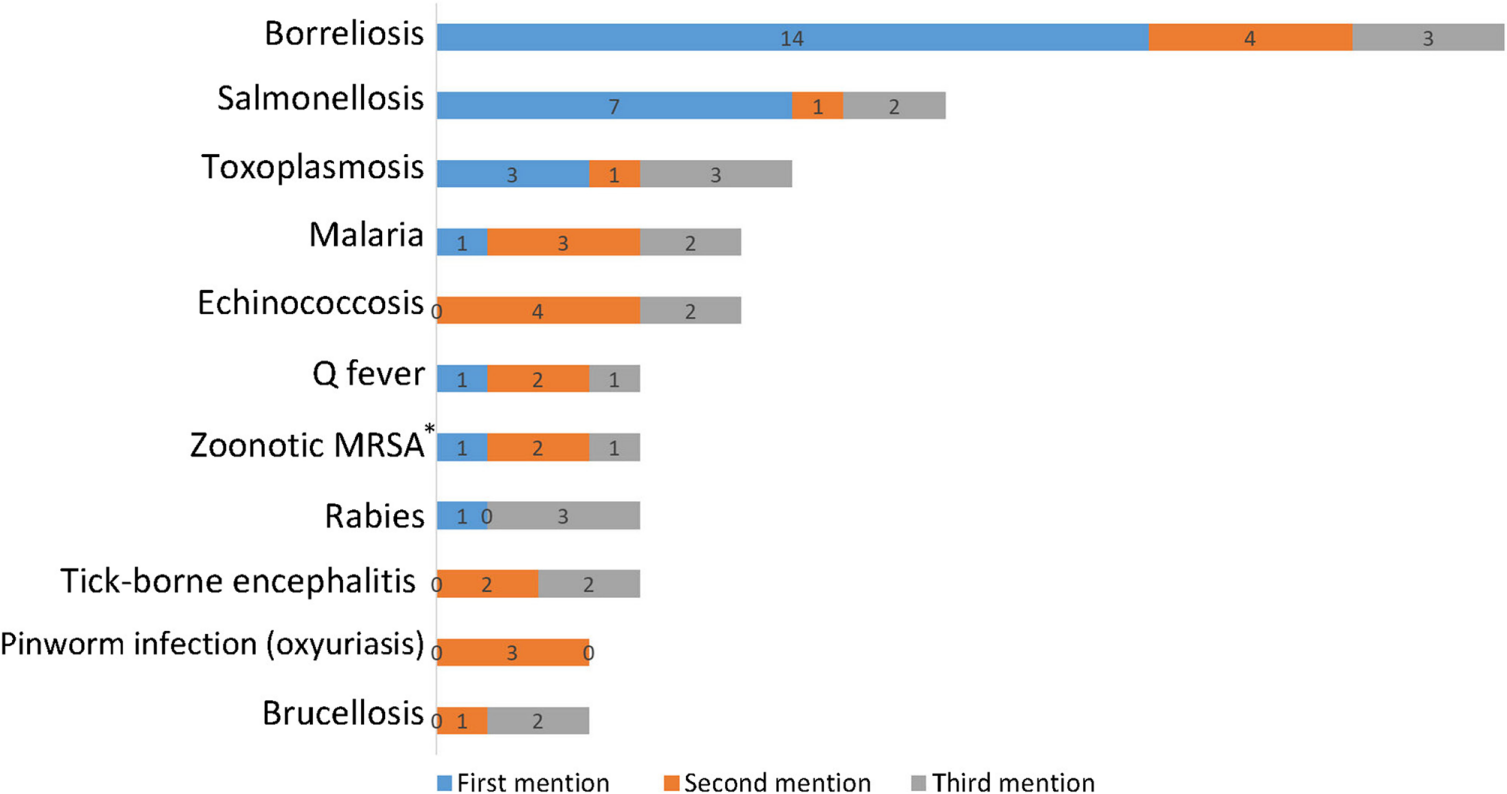
Zoonoses reported by respondents to have occurred in own practice (with at least three mentions across respondents)**. Notes: * Methicillin-resistant *Staphylococcus aureus*. ** By survey design, each respondent could report a maximum three zoonoses

Responding to the open question on which specific zoonoses they had ever encountered in their practice, to which respondents could list up to three, *N* = 38 generated 38 first, 33 s, und 27 third mentions, representing 32 infection types. As Fig. 1 shows, borreliosis (21 mentions), salmonellosis (10) und toxoplasmosis (7) occurred most often. All zoonoses with at least 3 mentions accounted for 73.5%.

**Fig. 2:**
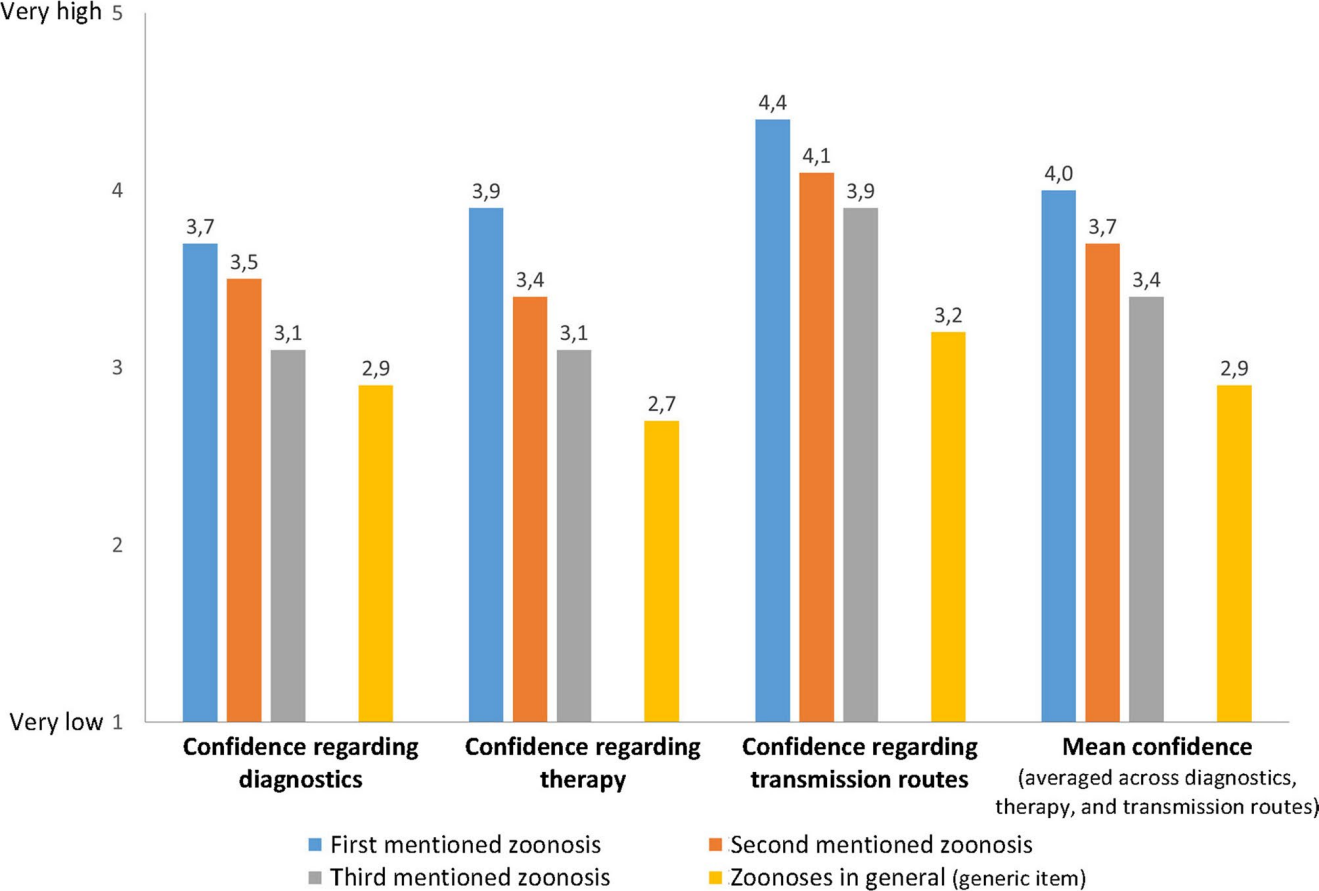
Confidence in own expertise regarding diagnostics, therapy, and transmission of zoonoses, for every zoonosis mentioned and zoonoses in general*,**. Notes: * Bars represent means (for results of statistical tests of differences, see Table 3). ** By survey design, each respondent could report a maximum of three zoonoses

Finally, participants’ mean ratings of their confidence regarding diagnostics, therapy, and transmission routes for the up to three self-reported zoonoses, and zoonoses in general (generic item), are shown in Fig. 2. Confidence numerically decreased from the first mentioned zoonosis to the generic item for all three issues, i.e., diagnostics, therapy, and transmission routes, and overall (averaged across issues). As Table 3(a) reveals, however, all differences between both the first vs. the second and the second vs. the third mentioned zoonoses do not show any statistical significance. In contrast, all comparisons between the self-reported zoonoses and the generic item were statistically significant (with two exceptions: zoonosis 3 regarding the issues of diagnostics and of therapy). Finally, differences across issues were statistically significant for “diagnostics vs. transmission routes” and “therapy vs. transmission routes” for all self-reported zoonoses and for the generic item, but never for the contrast “diagnostics vs. therapy” (see Table 3(b)).

**Table 3:** Confidence in own expertise regarding diagnostics, therapy, and transmission of zoonoses, for every zoonosis mentioned and zoonoses in general: results of statistical test for pairwise differences^*,**^

(a)	…Zoonosis 2	…Zoonosis 3	…Zoonoses in general
Confidence regarding diagnostics			
Zoonosis 1 vs…	t(29) = 0.76, *p* = 0.451 z = 0.66, *p* = 0.511	t(23) = 1.84, *p* = 0.079 z = 1.68, *p* = 0.092	t(35) = 4.94, *p* < 0.001 z = 3.86, *p* < 0.001
Zoonosis 2 vs…		t(23) = 0.85, *p* = 0.404 z = 0.74, *p* = 0.461	t(29) = 2.30, *p* = 0.029 z = 2.19, *p* = 0.028
Zoonosis 3 vs…			t(23) = 1.16, *p* = 0.258 z = 1.17, *p* = 0.241
Confidence regarding therapy			
Zoonosis 1 vs…	t(29) = 1.39, *p* = 0.174 z = 1.22, *p* = 0.223	t(23) = 1.75, *p* = 0.094 z = 1.59, *p* = 0.113	t(35) = 5.82, *p* < 0.001 z = 4.19, *p* < 0.001
Zoonosis 2 vs…		t(23) = 1.12, *p* = 0.276 z = 1.12, *p* = 0.264	t(29) = 2.99, *p* = 0.006 z = 2.68, *p* = 0.007
Zoonosis 3 vs…			t(23) = 1.97, *p* = 0.062 z = 2.01, *p* = 0.044
Confidence regarding transmission			
Zoonosis 1 vs…	t(29) = 1.75, *p* = 0.090 z = 1.65, *p* = 0.099	t(23) = 2.07, *p* = 0.050 z = 1.95, *p* = 0.051	t(34) = 9.94, *p* < 0.001 z = 5.06, *p* < 0.001
Zoonosis 2 vs…		t(23) = 0.97, *p* = 0.341 z = 0.93, *p* = 0.352	t(29) = 4.42, p *p* < 0.001 z = 3.40, *p* < 0.001
Zoonosis 3 vs…			t(23) = 3.33, *p* = 0.003 z = 2.81, *p* = 0.005
Overall confidence^***^			
Zoonosis 1 vs…	t(29) = 1.37, *p* = 0.182 z = 1.21, *p* = 0.228	t(23) = 2.04, *p* = 0.053 z = 2.02, *p* = 0.043	t(35) = 7.14, *p* < 0.001 z = 4.62, *p* < 0.001
Zoonosis 2 vs…		t(23) = 1.09, *p* = 0.284 z = 1.07, *p* = 0.285	t(29) = 3.61, *p* = 0.001 z = 3.02, *p* = 0.003
Zoonosis 3 vs…			t(23) = 2.42, *p* = 0.024 z = 2.17, *p* = 0.030

(b)	Diagnostics vs therapy	Diagnostics vs transmission	Therapy vs transmission
Zoonosis 1	t(35) = -1.07, *p* = 0.291 z = -1.07, *p* = 0.285	t(35) = -3.51, *p* = 0.001 z = -3.14, *p* = 0.002	t(35) = -2.76, *p* = 0.009 z = -2.51, *p* = 0.012
Zoonosis 2	t(29) = 0.42, *p* = 0.677 z = 0.28, *p* = 0.776	t(29) = -3.47, *p* = 0.002 z = -2.89, *p* = 0.004	t(29) = 3.88, *p* < 0.001 z = -3.26, *p* = 0.001
Zoonosis 3	t(23) = -0.01, *p* = 0.990 z = -0.01, *p* = 0.990	t(23) = -3.97, *p* < 0.001 z = -3.14, *p* = 0.002	t(23) = -3.10, *p* = 0.005 z = -2.66, *p* = 0.008
Zoonoses in general	t(35) = 1.64, *p* = 0.110 z = 1.60, *p* = 0.109	t(34) = -3.51, *p* = 0.001 z = -3.05, *p* = 0.002	t(34) = 3.72, *p* < 0.001 z = -3.14, *p* = 0.002

^*^t-statistics from t-tests for paired samples

^**^z-statistics from Wilcoxon signed-rank tests

^***^Mean of confidence ratings regarding diagnostics, therapy, and transmission paths

## Discussion

In a nutshell, this study indicates that zoonoses excluding COVID-19 clearly gained significance for German primary care practitioners up to the final phase of the COVID-19 pandemic. This is not only reflected in the ratings directly assessing perceived relevance of zoonoses, but may also be represented by the satisfactory survey response rate of 48.9%, which comes close to the mean found in an overview of 45 online physician surveys conducted in Europe (50.5% [32]). Zoonoses reported to occur most often in the practices of the participants were borrelioses, salmonelloses und toxoplasmoses. In actual patient care, however, zoonoses seem to play a lesser role or at least an occasion-related role only. This is also reflected in the lower levels confidence regarding diagnostics and therapy vs. transmission routes. At the same time, the relatively high confidence regarding zoonoses which have occurred in one’s own practice points to potentials for primary care: given practitioners have experience with a zoonosis, they can be confident in its regard (“practice makes perfect”). This may be transferable to more rare zoonoses for which effective care is important for patients as well.

Limitations of this study include the survey response rate of 48.9%. While as noted it was satisfactory when compared to empirical averages [32], it implies that more than half of the resident primary care practitioners in the district did not take part, which adds– besides item nonresponse– to survey error. However, the share of female respondents (41.2%) did not differ critically from that in the population (37.5%), and the residential area distribution (55.9% urban) resembles the proportion of population living in independent communities (51.5%; data accessed from Statistical Office of Lower Saxony at https://www.statistik.niedersachsen.de/startseite/ on December 2, 2024). Thus, the sample represented in this survey does seem to reach sufficient representativeness of the resident primary care practitioners in the district. At the same time, a similar assertion is not possible for the representativeness of the district of Hameln-Pyrmont for Germany. According to the German Federal Office for Building and Regional Planning, it represents a rural district with signs of urbanization (https://www.bbsr.bund.de/BBSR/DE/forschung/raumbeobachtung/Raumabgrenzungen/deutschland/kreise/siedlungsstrukturelle-kreistypen/kreistypen.html). Thus, further studies will have to replicate the survey design in other types of settlement geographical, e.g. metropolitan areas. Finally, the sample is small (*N* = 43 survey participants, *N* = 34 with complete data), and thus all analyses primarily have an explorative character with the aim to start to fill the gaps of knowledge on awareness and handling of zoonoses in primary care in Germany.

Keeping these limitations in mind, first it can be stated that strong salience of borreliosis evident in this survey is consistent with the focus of previous studies on Lyme disease [4–24]. Second, the results on confidence regarding diagnostics and therapy on the one hand indicate existing capacities in terms of relatively high confidence regarding salient zoonoses, i.e., those mentioned by the practitioners. That is to say, further scrutiny may focus on the cognitive, emotional and ethical domains of this confidence regarding zoonotic diseases care in order to build on and improve practitioners’ awareness [29]. On the other hand, room for improvement is indicated by the confidence ratings being lower than regarding the transmission of zoonotic. Here, tools for diagnosing and managing zoonotic infections such as the algorithm developed for general practice in Australia [33] may represent one pathway forward.

## Conclusions

In sum, awareness of uncertainties regarding zoonoses among primary care practitioners may serve as triggers to intensify both continuing medical education and cooperation between doctors of medicine and veterinarians, which seem to be rarely pursued in real-life outpatient primary care. One starting point could be the issue of pets, which the vast majority of the practitioners in this survey (94.3%) report to make a subject of in the course of caring for their patients at least on occasion. This could contribute both to the implementation of the emerging One Health strategy [34] and the empowerment of primary care practitioners further develop their role in zoonosis management initially outlined 60 years ago [35].

## Supplementary Information

Supplementary Material 1.

## Data Availability

The data set is available from the corresponding author upon reasonable request.

## Abbreviations

COVID-19: Coronavirus disease 2019
Destatis: German Federal Statistical Office
SARS: Severe acute respiratory syndrome
