# Supplementary material for: Resident primary care practitioners’ awareness and handling of zoonotic diseases: an explorative online survey in the Hameln-Pyrmont district, Lower Saxony, Germany
Source: BMC Prim Care. 2025 Aug 8;26:249. doi: 10.1186/s12875-025-02918-7 (PMC12333134; doi:10.1186/s12875-025-02918-7)
Supplement: Supplementary file 1 — Supplementary Material 1. [file 12875_2025_2918_MOESM1_ESM.pdf]

Hardcopy Version of the Online Survey Questionnaire of AVZA-study (English translation of the German original: The authors)

Supplementary Material to doi: 10.1186/s12875-025-02918-7

Dear colleague,

Zoonotic diseases are very heterogeneous. This can pose a considerable challenge in diagnosis and treatment. It is therefore important that medical doctors are sufficiently familiar with the pathogens and diseases that can be transmitted between animals and humans.

This applies first and foremost to primary care practitioners, who are undoubtedly the primary and most important point of contact for patients in this context.

Although zoonoses are on the rise, research findings in this area are often only hesitantly put into practice. The severe consequences that zoonotic diseases can have were made clear to the global community by the COVID-19 pandemic.

The present study is conducted to clarify the extent to which zoonoses are known, perceived and treated by general practitioners:

"Resident primary care practitioners' awareness and handling of zoonotic diseases: an explorative online survey in the Hameln-Pyrmont district, Lower Saxony, Germany"

The findings are intended to show ways of better meeting the need for information on zoonoses in primary care.

Since there is currently a great risk that the current focus on the SARS-CoV-2 virus will lead to overlook the numerous other zoonotic pathogens, this study was designed in such a way that the survey largely excludes the consideration of COVID-19.

If you would like to comment on any of the following questions, please use the free text field at the end of the questionnaire.

We appreciate your opinion and thank you in advance for your participation!

Michael Scheider,
Hannover Medical School, Center for Public Health

next page

I. DATA PROTECTION DECLARATION:

Study information and informed consent for the study "Resident primary care practitioners' awareness and handling of zoonotic diseases: an explorative online survey in the Hameln-Pyrmont district, Lower Saxony, Germany"

Participation in this anonymous survey is voluntary. Non-participation has no disadvantages for you.

The e-mail addresses were determined via the Association of Statutory Health Insurance Physicians of Lower Saxony following prior approval by the Lower Saxony Ministry for Social Affairs, Health and Equality. It is not possible to trace your e-mail address back to the online questionnaire or vice versa.

Your e-mail address will be completely deleted after the end of the survey.

Once you have completed the online questionnaire, your details will be transferred to the MHH Information Technology (MIT) server in a non-personally identifiable form, where they will be stored and processed in accordance with data protection laws. As long as the questionnaire has not been sent, participation in the study can be terminated at any time without giving reasons.

Please note that this study does not store any personal identifying data together with the answers and that it is not possible to assign specific answers to you personally, so that your data cannot be deleted retrospectively.

Data controller: Prof. Dr. Thomas von Lengerke, Hannover Medical School, Center for Public Health Care (OE 5430), Carl-Neuberg-Strasse 1, 30625 Hannover.

If you have any questions or if you believe that your personal data is not being processed lawfully, you can contact the MHH Data Protection Officer: MHH Data Protection Officer, OE 0007, Carl- Neuberg-Straße 1, 30625 Hannover.

You have the right to lodge a complaint with the supervisory authority if you believe that your personal data is being processed unlawfully. The address of the supervisory authority responsible for the MHH is: State Commissioner for Data Protection for Lower Saxony, Prinzenstraße 5, 30159 Hannover.

After completion of the study, the data from the completed questionnaire will be stored for ten years in accordance with the guidelines of good scientific practice and will be irrevocably deleted after this period has expired.

I hereby agree to participate in the survey. I understand that my participation in this study is voluntary and that I can withdraw my consent at any time without giving reasons and without any disadvantages for me.

O Yes

O No

Filter: If "No", questionnaire ends, option to return if clicked fails → end of survey

Do you not want to participate in this survey? In this case, the survey ends here. Thank you very much.
You may now close the browser window. If you do wish to participate, please click on this button:

change consent

Filter: If "Yes", then continue with next question

2. Are you aware of at least one zoonosis?

O Yes

O No

Filter: If "No", questionnaire ended, option to return if click fails → End of survey

Since further processing of the questionnaire requires awareness of at least one zoonosis, the survey is terminated at this point. Thank you very much for your participation. You may now close the browser window. If you do know of a zoonosis, click the following button:

I am aware of a zoonosis

Filter: If clicked, then continue with next page

previous page *next page*

3. How do you rate the relevance of the topic of zoonoses for your practice in the period before the start of the Covid-19 pandemic?

1 2 3 4 5

Not relevant at all ◯ ◯ ◯ ◯ ◯ Very relevant

4. How do you rate the relevance of the topic of zoonoses for your practice in the period after the start of the Covid-19 pandemic?

Not relevant at all ◯ ◯ ◯ ◯ ◯ Very relevant

5. Were zoonoses explicitly taught in your medical studies?

◯ Yes

◯ No

◯ I do not remember

Please be reminded that the following questions all relate to zoonoses EXCEPT COVID-19!

6. Have you attended any educational and/or training courses on zoonoses in the last 10 years?

◯ Yes

◯ No

◯ I do not remember

7. How many educational and/or training courses on zoonoses have you completed so far?

◯ 1-2

◯ 3-5

◯ more than five

previous page *next page*

MHH Medizinische Hochschule Hannover

8. Now please name up to 3 zoonoses that you have already dealt with in your practice:

1. Zoonosis [ ]

2. Zoonosis [ ]

3. Zoonosis [ ]

Filter: If a zoonosis is entered, continue with next items

The following three questions refer to the zoonoses you mentioned (presented for each of the mentioned zoonoses)!

9. How would you rate your confidence in regard to diagnosing the zoonoses you mentioned in question 8?

	1	2	3	4	5	
very low	○	○	○	○	○	very high

10. How would you rate your confidence in regard to treating the zoonoses you mentioned in question 8?

	1	2	3	4	5	
very low	○	○	○	○	○	very high

11. How would you rate your confidence in regard to the transmission routes of the zoonoses you mentioned in question 8?

	1	2	3	4	5	
very low	○	○	○	○	○	very high

previous page *next page*

The following questions relate to zoonoses in general EXCEPT COVID-19!

12. How would you rate your confidence in regard to diagnosing zoonoses in general?

	1	2	3	4	5	
very low	○	○	○	○	○	very high

13. How would you rate your confidence in regard to treating zoonoses in general?

	1	2	3	4	5	
very low	○	○	○	○	○	very high

14. How would you rate your confidence in regard to transmission routes of zoonoses in general?

	1	2	3	4	5	
very low	○	○	○	○	○	very high

previous page *next page*

15. How do you treat zoonosis in your practice?

- O usually yourself
- O depending on the situation (e.g. type of pathogen/severity of symptoms/therapy for the patient)
- O never oneself (e.g. only referral)

16. Please estimate: How often do zoonoses occur in your family practice?

[ ] times per year

17, Do you ever ask a patient about pet ownership?

- O Yes
- O No

18. If yes: On what occasions are you most likely to ask patients about pet ownership? (multiple choices possible)

- O at first admission
- O in case of health problems, i.e., symptom-driven
- O at other occasions (e.g., preventive measures)

previous page *next page*

19. Please estimate: What percentage of your patients are pet owners?

[ ] %

20. Please estimate: How many of your patients have regular contact with ...

...pets? [ ] %

...wild animals? [ ] %

...farm animals? [ ] %

21. Do you counsel patients on the prevention of zoonotic diseases in your practice?

O Yes, often

O Sometimes

O No, never

22. Do you counsel patients on the treatment of zoonotic diseases in your practice?

O Yes, often

O Sometimes

O No, never

23. Is information material on zoonoses available in your practice?

O Yes

O No

24. Have you ever worked with a veterinarian in the context of zoonosis treatment?

O Yes

O No

25. Are you aware of where you can obtain up-to-date information of zoonoses?

O Yes

O No

26. Do you know the term One Health?

O Yes

O No

27. What gender are you?

- O Male
- O Female
- O Diverse

28. The results of this survey are analyzed overall and stratified for different age groups. How old are you?

- O >= 30
- O 31 – 40
- O 41 – 50
- O 51 – 60
- O > 60

29. How many years have you been working as a general practitioner?

- O up to 10 years
- O 11 – 20 years
- O more than 20 years

30. What kind of practice do you work in?

- O Single practice
- O Group or shared practice

31. In which kind of residential area is your practice located?

- O Urban
- O Rural

32. How many patients are treated in your practice per quarter?

- O 500 – 750
- O 751 – 1000
- O 1001 – 1250
- O 1251 – 1500
- O 1501 – 1750
- O > 1750

That brings us to the end of our questionnaire. Do you have any further suggestions regarding the topic or the survey itself? (This is an anonymous survey, so please avoid personally identifying information).

Medizinische Hochschule Hannover

Thank you for your participation! Your answers have been saved, you can now close the browser window.
